# Prior culture-guided prediction of antibiotic susceptibility in recurrent respiratory tract infections: a retrospective cohort analysis

**DOI:** 10.3389/fcimb.2025.1715986

**Published:** 2026-01-06

**Authors:** Shiyu Li, Wenxia Zhang, Hanwen Ma, Xiang Gao, Weian Yuan, Min He

**Affiliations:** 1 Shuguang Hospital Affiliated to Shanghai University of Traditional Chinese Medicine, Shanghai, China; 2 Department of Clinical Laboratory, Zhoupu Hospital, Shanghai University of Medicine and Health Sciences, Shanghai, China

**Keywords:** respiratory tract infections, recurrence, antibiogram, antibiotic resistance, Bayesian prediction

## Abstract

**Background:**

Recurrent respiratory tract infections (RRTIs) are a serious problem in older adults, necessitating appropriate early empiric antibiotic therapy. However, rising antimicrobial resistance complicates empiric treatment selection. Although prior culture results can offer guidance for empiric decisions, little data exist to quantify their predictive value in RRTIs. Therefore, we constructed a respiratory antibiogram and assessed Bayesian metrics of prior gram-negative isolates for predicting subsequent resistance or susceptibility in this population.

**Method:**

A retrospective cohort included hospitalized RRTI patients (defined as RTIs occurring ≥2 times in 6 months or ≥3 times in 12 months). Patient-specific antibiograms were constructed. A Bayesian prevalence-calibrated estimation was used to assess the predictive validity of prior respiratory cultures for subsequent antimicrobial susceptibility in paired isolates. Sensitivity (Sen), specificity (Spec), Bayesian positive predictive value (PPV), negative predictive value (NPV), and resistance prevalence were calculated for 24 antibiotics, with bootstrap 95% confidence intervals. Analyses were restricted to bacterial pathogens.

**Result:**

We included 463 visits from 160 unique patients, with a median age of 70. Pathogen distribution revealed Gram-negative bacteria (89.50%) and Gram-positive bacteria (10.50%). Antibiogram results showed that *Pseudomonas aeruginosa* exhibited >30% resistance to 10 antibiotics, while *Klebsiella pneumoniae* exhibited >35% resistance to 16 antibiotics. Prior cultures had good predictive value (>70%) for detecting future resistance to 22 antibiotics, with ciprofloxacin, levofloxacin, cefuroxime, cefotaxime, ampicillin, ampicillin/sulbactam, and ceftriaxone showing excellent predictive values (≥85%). Additionally, prior cultures had excellent predictive value (≥85%) for detecting future susceptibility to imipenem, amikacin, tobramycin, meropenem, minocycline, and tigecycline.

**Conclusion:**

Considerable antibiotic resistance was detected among isolates in patients with RRTIs. Using a prior culture as a guide can enhance the probability of selecting an effective empirical agent.

## 1 Introduction

Respiratory tract infections (RTIs) are one of the major global health problems and are common across all age groups; however, they pose an increased risk in older populations due to age-related physiological decline and the high prevalence of chronic comorbidities (Furman et al., 2021; HespanholBarbara, 2020; Liu et al., 2023; Torres et al., 2021; Tsoumani et al., 2023). Specifically, RRTIs constitute a serious clinical problem among older adults, associated with significant morbidity, mortality, and economic cost (Dang et al., 2015; Henig and Kaye, 2017; Shi et al., 2020; Shiroshita et al., 2022). According to the 2019 Global Burden of Disease study, lower respiratory tract infections, including pneumonia and bronchiolitis, affected 489 million people globally and were responsible for >2.49 million deaths, with mortality highest among patients aged >70 years (1.23 million deaths).

Bacterial pathogens represent one of the most prevalent etiological agents of RTIs, with antibiotic regimens remaining the cornerstone of clinical management (Gil and Webb, 2020; Lieberman and Lieberman, 2003; Raghav et al., 2022). Early empiric antibiotic therapy is critical for infection control, particularly in older patients with RRTIs (Martin-Loeches et al., 2022). Observational studies indicate that early administration of appropriate empiric antibiotic therapy is associated with reduced morbidity and mortality, shorter hospital stays, and lower healthcare costs across multiple infections (Akinosoglou et al., 2021; Bassetti et al., 2020; Kadri et al., 2021; Nie et al., 2018). Empiric therapy refers to the initial antibiotic treatment chosen before the causative pathogen is definitively identified and antimicrobial susceptibility test results become available. Antibiotic stewardship programs currently face the dual challenge of selecting effective empirical regimens while minimizing antimicrobial resistance development and patient harm. However, the rising prevalence of antimicrobial resistance complicates empiric therapy decisions and increases the risk of inappropriate treatment (Strich et al., 2020). Personalized treatment is essential due to varying patient risks for antibiotic-resistant organism infections. Prior culture results can provide valuable guidance for empiric therapy decisions, especially for patients with recurrent infections (Elligsen et al., 2021; Kim et al., 2023; Valentine-King et al., 2024). Nevertheless, research examining the predictive value of prior cultures remains limited in RRTIs.

Here, we propose a personalized approach for patients with RRTIs, through the development of patient-specific antibiograms and the evaluation of the predictive value of prior cultures for subsequent antibiotic resistance and susceptibility.

## 2 Materials and methods

### 2.1 Study design

We retrospectively extracted data from the RuiMei Laboratory Information System database on hospitalized patients with RRTIs at Shuguang Hospital affiliated to Shanghai University of Traditional Chinese Medicine between January 1, 2019, and January 1, 2023. Briefly, we included inpatients aged ≥18 years with recurrent RTIs documented in medical records as occurring ≥2 times in 6 months or ≥3 times in 12 months, and with at least two bacterial infections confirmed by culture during these episodes (Braido et al., 2014; Lapi et al., 2024; Van Kessel et al., 2014). Each patient was eligible for inclusion only once. For this study, “respiratory tract infections” specifically referred to bacterial lower respiratory tract infections. The study was approved by the Ethics Committee of Shuguang Hospital affiliated to Shanghai University of Traditional Chinese Medicine (protocol number TRQYQ2023) and a waiver of consent was granted for this database study.

### 2.2 Antibiogram construction

The respiratory specimens for bacterial cultures comprised sputum, lavage fluid, and catheter samples. Susceptibility data extraction was restricted to bacterial species with ≥30 isolates, to ensure robust statistical analysis. For an accurate representation of antibiotic susceptibilities in patients with RTIs at any one time, we used all isolates obtained from each patient. In our antibiogram analyses, bacterial isolates exhibiting intermediate susceptibility were classified as resistant to align with conservative clinical decision-making practices. Antibiotic susceptibility testing was performed according to Clinical and Laboratory Standards Institute guidelines.

Fungal isolates (predominantly Candida spp.) were excluded from the main prediction analyses because they typically represent colonization in respiratory specimens, which rarely warrants antifungal therapy per IDSA/ESCMID guidance, and because of a lack of valid pairs. Detailed antifungal susceptibility data for these isolates are provided in the **Supplementary Materials** for reference only; these findings should not guide empiric therapy in the absence of evidence of invasive disease (Boonsilp et al., 2021; Seyoum et al., 2020; Makled et al., 2024).

Bacterial etiology was considered confirmatory of infection, rather than colonization, when a pathogenic bacterium was cultured from a high-quality lower respiratory tract specimen with a quantitative culture yielding ≥10^4^ CFU/mL and when this finding was accompanied by clinical signs of infection (e.g., fever, leukocytosis, elevated C-reactive protein or procalcitonin) and radiographic evidence of new or progressive pulmonary infiltrates.

### 2.3 Test metrics

**Figure 1:**
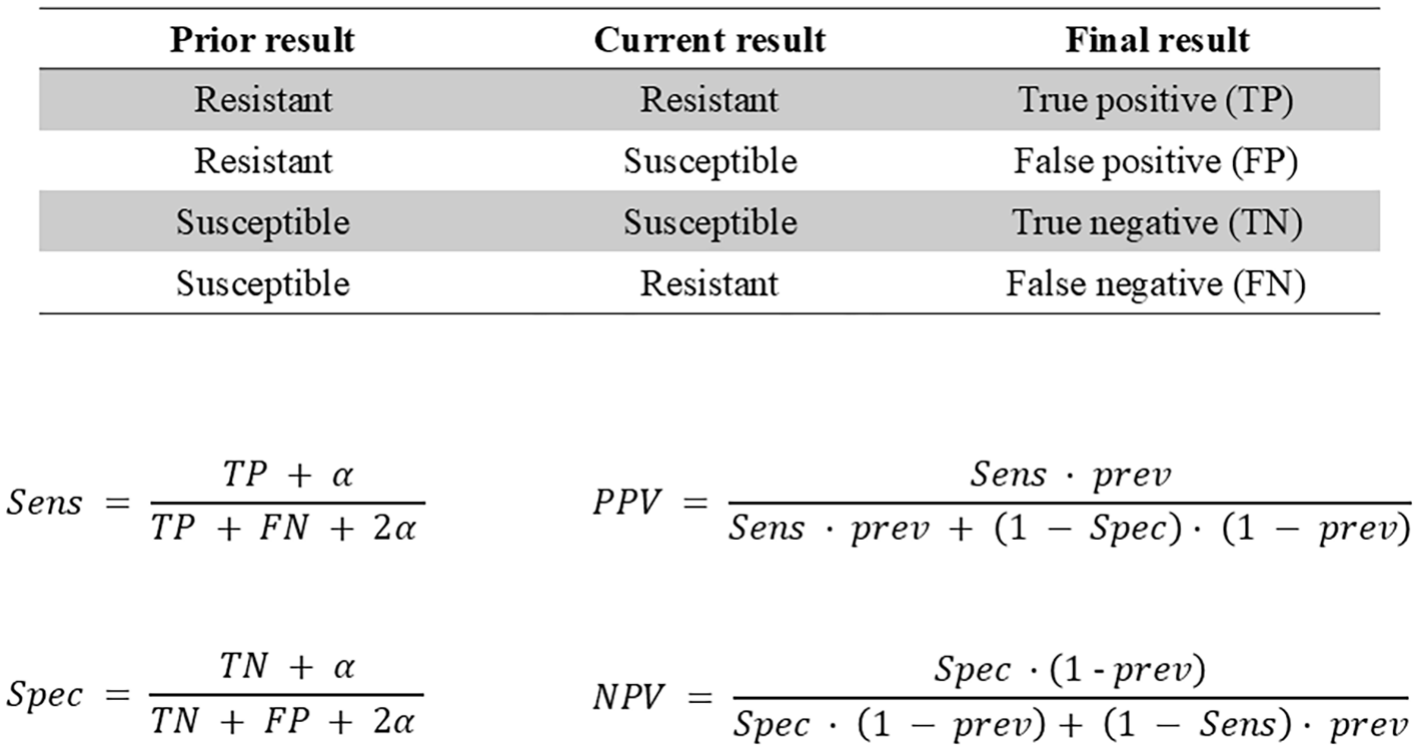
Test metric derivation used to calculate sensitivity (Sen), specificity (Spec), traditional positive predictive value (PPV), negative predictive value (NPV), and Bayes’ PPV and NPV. Prev indicates prevalence, α indicates Laplace smoothing.

To evaluate the predictive capacity of a prior culture on subsequent antimicrobial resistance/susceptibility, we considered that the prior culture from a single patient served as the “test”, while the current culture from the same individual served as the “gold standard” (**Figure 1**). We assessed concordance between paired gram-negative isolates derived from the same patient. When multiple antimicrobial susceptibility results were available for the same strain during a single hospitalization, we used the first result for comparative analysis, as it reflected the initial infection status. If a patient had multiple gram-negative organisms from the same visit, paired comparative analyses were conducted separately for each organism against either the prior culture or the subsequent culture.

We calculated sensitivity, specificity, Bayes’ positive predictive value (PPV), and negative predictive value (NPV) using standard formulas (**Figure 1**). Bayesian formulae, which incorporate pretest probability or prevalence of resistance, are particularly suitable for paired culture analyses because PPV increases with higher resistance prevalence while NPV decreases. To ensure our results accurately reflected the test’s performance across the entire sample, we used the antibiotic resistance prevalence from the entire study population (including all patients with gram-negative organisms) as the prior probability, rather than limiting it to the subset with paired cultures. This approach provides a more reliable assessment of the test’s effectiveness based on the resistance patterns observed in our complete datasets.

At the patient level, paired prior and subsequent cultures were conceptually framed within a Bayesian prevalence-calibrated estimation framework. In this framework, sensitivity and specificity derived from paired isolates represent the empirical likelihood of resistance, while the overall institutional resistance prevalence serves as the prior probability. These components were integrated using standard Bayesian updating to generate posterior probabilities (Bayes’ PPV and NPV), thereby enabling prevalence-aware interpretation of how prior culture results inform empiric antimicrobial decision-making.

This study employed patient-level (cluster) bootstrap resampling with 10,000 replicates to calculate 95% confidence intervals for sensitivity (Sen), specificity (Spec), Bayesian positive/negative predictive values (PPV/NPV), and resistance rates. To enhance the robustness of the predictive approach, Laplace smoothing (α) was applied with three α values (0.1, 0.5, 1.0) chosen based on sample size categories, mitigating potential biases from small samples or extreme data (Harrell et al., 1996; Steyerberg et al., 2001). We used α = 0.1 for well-powered strata (≥30 observations), α = 0.5 for moderate sample sizes (10–29 observations), and α = 1.0 when any count was zero or when a component contained <10 observations. The random number generator seed was fixed at 42 to ensure reproducibility. Analysis code and environment files are available from the corresponding author upon reasonable request.

## 3 Result

**Figure 2:**
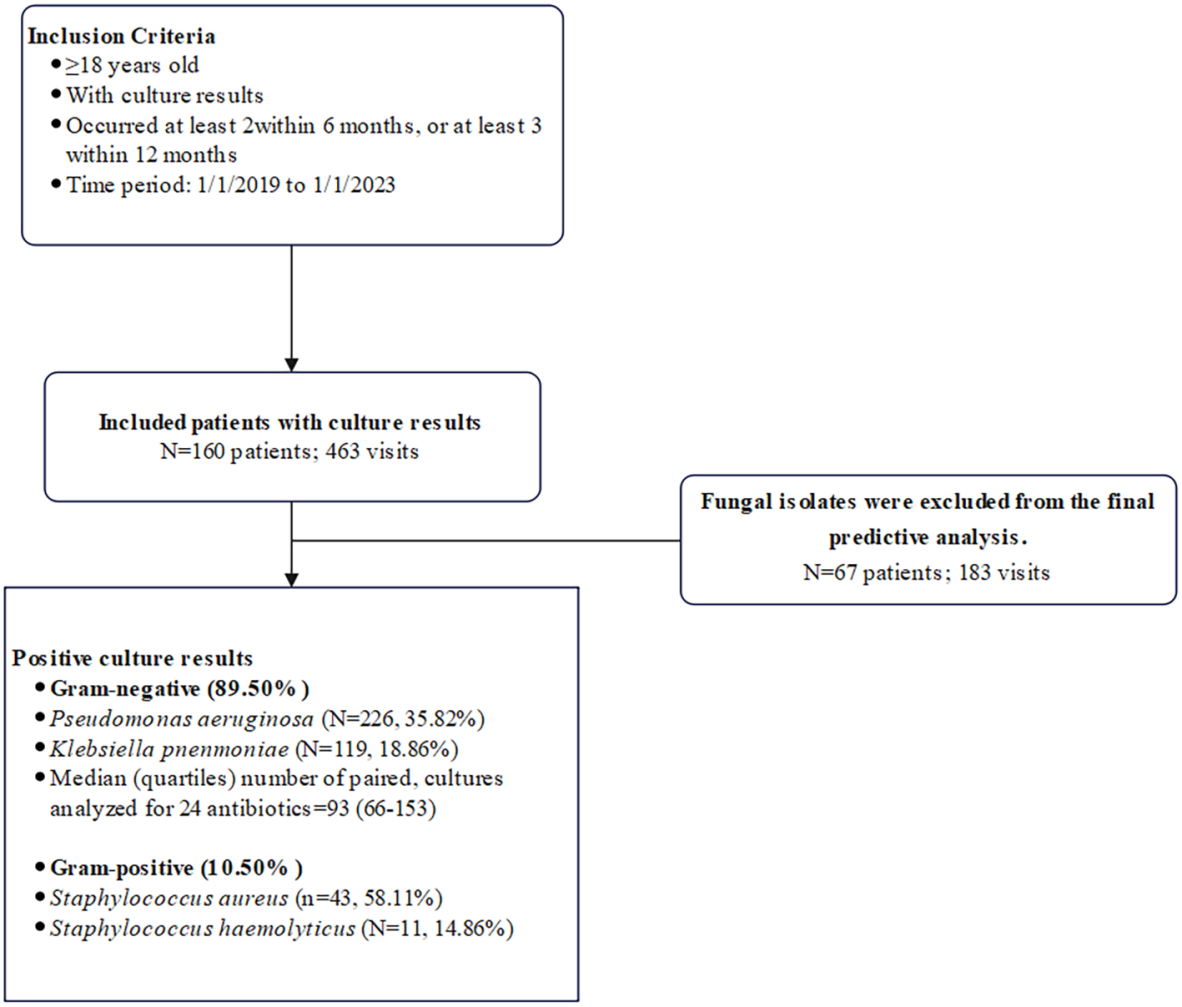
Flow chart depicting patient and visit inclusion criteria, overview of bacterial culture results, and breakdown of bacterial cultures eligible for antibiogram and paired culture analysis among antibiotics evaluated.

**Figure 3:**
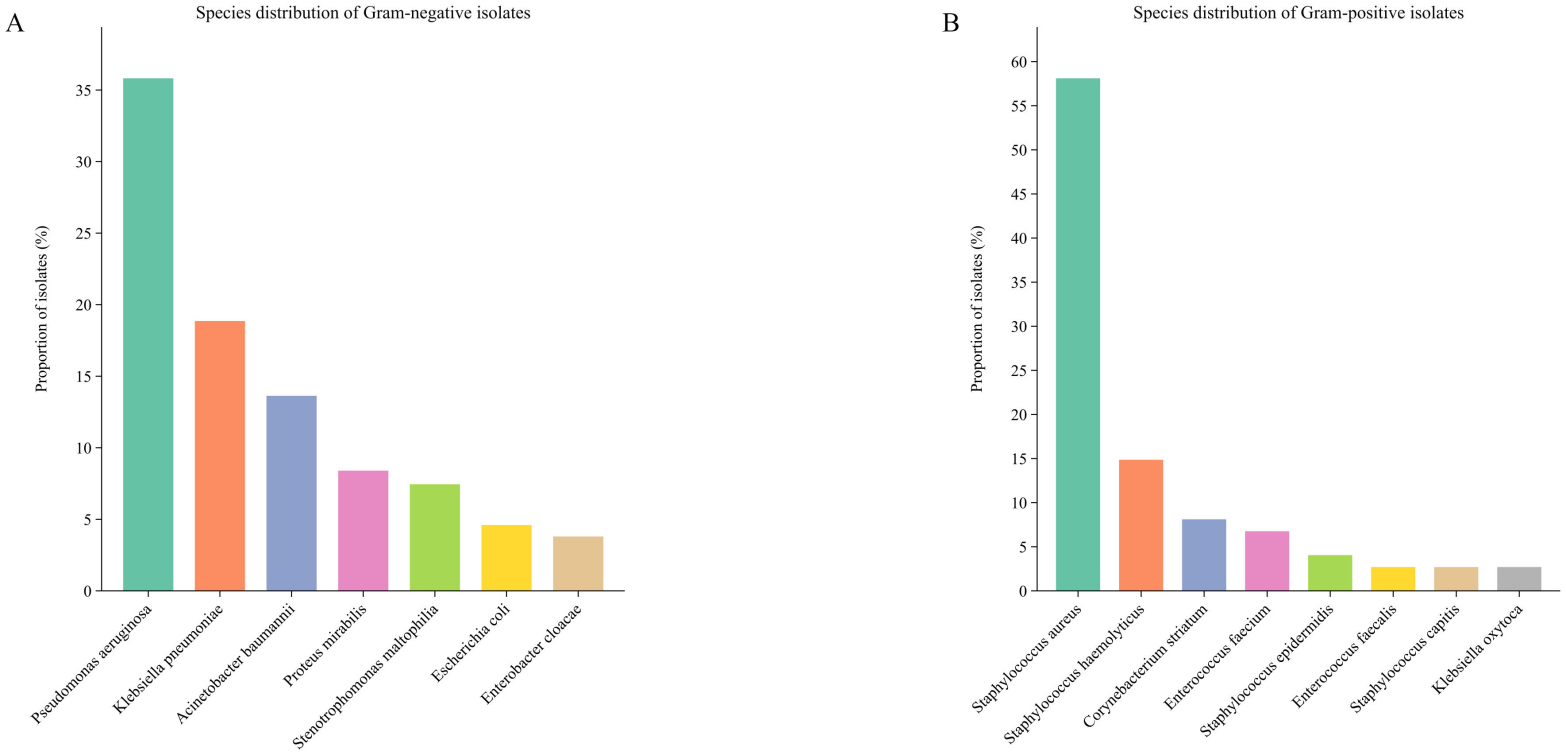
Respiratory tract organism prevalence among 705 organisms from 280 RRTI hospitalization visits. **(A)** Species distribution of Gram-negative bacteria. **(B)** Species distribution of Gram-positive isolates.

After applying exclusion criteria, our study population consisted of 160 hospitalized patients (including 32 ICU patients, 5 of whom underwent endotracheal intubation) and represented 463 hospitalization visits for RRTIs (**Figure 2**). Among 463 hospitalization visits with a culture, 193 (41.2%) tested positive for ≥ 2 pathogens. A total of 1,138 pathogens (54 species) were isolated from all samples. Among the bacterial isolates, gram-negative bacteria accounting for 89.50% (N = 631) and gram-positive bacteria for 10.50% (N = 74). *Pseudomonas aeruginosa* (N = 226, 35.82%) was the predominant gram-negative isolate, followed by *Klebsiella pneumoniae* (N = 119, 18.86%), *Acinetobacter baumannii* (N = 86, 13.63%), *Proteus mirabilis* (N = 53, 8.40%), and *Stenotrophomonas maltophilia* (N = 47, 7.45%) (**Figure 3A**). *Staphylococcus aureus* (n=43, 58.11%) and *Staphylococcus haemolyticus* (N = 11, 14.86%) were the most frequent gram-positive isolates (**Figure 3B**). Of the 463 visits with gram-negative organisms; the median number of isolates tested for antibiotic susceptibility across 24 antimicrobials was 170 (IQR 111-254). The patient characteristics are listed in **Table 1**. The median age of the study population was 70 years (IQR 63-77), and 68.75% were male. These patients had a median of 2 (IQR 2-3) hospitalization visits throughout the study period, with a median of 103 (IQR 49-268) days between visits.

**Table 1:** Characteristics of the overall study population.

Patient characteristics	Patients with a GN organism (N=93)		All patients (N=160)	
	value	% / quartiles	value	% / quartiles
Age, median	70	(63-77)	70	(63-77)
Male	63	67.74	110	68.75
Female	30	32.26	50	31.25
Visits per patient, median	2	(2-4)	2	(2-3)
Visit characteristics	(N=280)		(N=463)	

### 3.1 Antibiogram results

**Figure 4:**
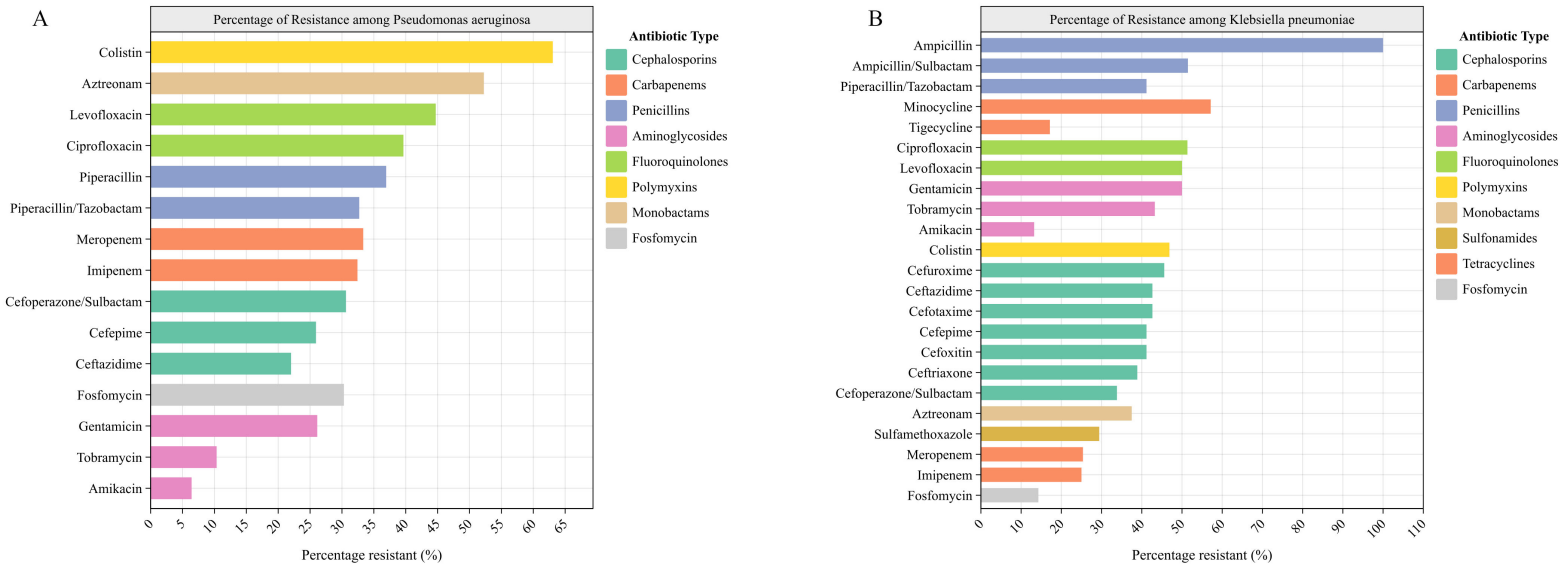
Antibiogram. **(A)***Pseudomonas aeruginosa*, **(B)***Klebsiella pneumoniae*. Antibiotics are grouped and their bars are colored according to antibiotic category.

Our *Pseudomonas aeruginosa* antibiogram showed that the highest levels of resistance were to colistin (63.06%, 95% CI: 54.05%-72.07%), aztreonam (52.25%, 95% CI: 42.34%-61.26%), levofloxacin (44.71%, 95% CI: 34.12%-55.29%), ciprofloxacin (39.64%, 95% CI: 30.63%-48.65%), piperacillin (36.94%, 95% CI: 27.93%-45.95%), meropenem (33.33%, 95% CI: 24.32%-42.34%), piperacillin-tazobactam (32.71%, 95% CI: 24.30%-42.06%), imipenem (32.43%, 95% CI: 24.32%-41.44%), cefoperazone-sulbactam (30.63%, 95% CI: 22.52%-39.64%), fosfomycin (30.30%, 15.15%-45.46%), gentamicin 26.13% (95% CI: 18.02%-34.23%), cefepime (25.93%, 95% CI: 17.59%-34.26%), ceftazidime (22.02%, 95% CI: 14.68%-30.28%). The lowest resistance was observed to amikacin (6.42%, 95% CI: 1.84%-11.01%) and tobramycin (10.34%, 95% CI: 4.60%-17.24%) (**Figure 4A**).

The *Klebsiella pneumoniae* isolates exhibited marked resistance, with ampicillin demonstrating universal resistance (100%). High-level resistance (>40%) was observed to 13 agents, including minocycline (57.14%, 95% CI: 28.57%-78.57%), ampicillin-sulbactam (51.47%, 95% CI: 39.71%-63.24%), ciprofloxacin (51.35%, 95% CI: 35.14%-67.57%), levofloxacin (50.00%, 95% CI: 38.24%-61.77%), gentamicin (50.00%, 95% CI: 38.24%-61.77%), colistin (46.88%, 95% CI: 34.38%-59.38%), cefuroxime (45.49%, 95% CI: 33.82%-57.35%), tobramycin (43.24%, 95% CI: 27.03%-59.46%), cefotaxime and ceftazidime (42.65%, 95% CI: 30.88%-54.41%), piperacillin-tazobactam, cefoxitin and cefepime (41.18%, 95% CI: 29.41%-52.94%), Ceftriaxone (38.89%, 95% CI: 22.22%-55.56%), aztreonam (37.50%, 95% CI: 12.50%-62.50%), cefoperazone-Sulbactam (33.82%, 95% CI: 23.53%-45.59%), sulfamethoxazole (29.41%, 95% CI: 19.12%-39.71%), meropenem (25.37%, 95% CI: 14.93%-35.82%), and imipenem (25.00%, 95% CI: 14.71%-35.29%) showed moderate resistance. However, three agents exhibited lower resistance: amikacin (13.24%, 5.88%-22.06%), fosfomycin (14.29%, 3.57%-28.57%), and tigecycline (17.14%, 5.71%-31.43%) (**Figure 4B**).

### 3.2 Prior culture predictability among gram-negative bacteria

We reported test metric data for 24 RTI-relevant antimicrobial agents (**Table 2**). Among these 24 agents, the median interval between paired cultures was 103 days. Additionally, test metric data for Candida albicans were excluded, as only sensitivity data were available (no culture pairs met the criteria for classification as true/false positives or negatives).

**Table 2:** Test metric results from comparing paired, gram-negative organisms by antibiotic.

Antimicrobial agent	paired isolates	Resistance, %^a^	Sensitivity (95% CI)^b^	Bayes' PPV (95% CI)^c^	Specificity (95% CI)^d^	Bayes' NPV (95% CI)^e^
Ceftazidime	169	41.22	0.66 (0.54-0.76)	0.75 (0.67-0.84)	0.85 (0.77-0.92)	0.78 (0.73-0.83)
Cefepime	152	42.63	0.68 (0.55-0.79)	0.74 (0.64-0.84)	0.82 (0.72-0.90)	0.78 (0.71-0.84)
Imipenem	155	34.51	0.73 (0.59-0.84)	0.73 (0.63-0.85)	0.86 (0.78-0.93)	0.86 (0.80-0.91)
Cefoperazone/Sulbactam	170	34.52	0.58 (0.45-0.69)	0.70 (0.60-0.82)	0.87 (0.81-0.93)	0.80 (0.75-0.85)
Piperacillin/Tazobactam	164	43.17	0.68 (0.55-0.79)	0.75 (0.66-0.85)	0.83 (0.75-0.91)	0.77 (0.71-0.84)
Amikacin	149	17.93	0.58 (0.39-0.73)	0.73 (0.58-0.91)	0.95 (0.91-0.99)	0.91 (0.88-0.94)
Tobramycin	86	31.14	0.80 (0.63-0.93)	0.83 (0.71-0.94)	0.93 (0.85-0.98)	0.91 (0.85-0.97)
Ciprofloxacin	117	55.67	0.80 (0.69-0.89)	0.89 (0.82-0.95)	0.88 (0.78-0.95)	0.78 (0.69-0.86)
Levofloxacin	149	57.81	0.79 (0.70-0.87)	0.89 (0.83-0.95)	0.87 (0.78-0.95)	0.75 (0.68-0.83)
Colistin	132	55.17	0.75 (0.63-0.84)	0.76 (0.68-0.84)	0.71 (0.57-0.82)	0.70 (0.60-0.79)
Piperacillin	70	36.94	0.45 (0.24-0.65)	0.49 (0.31-0.66)	0.73 (0.57-0.85)	0.69 (0.60-0.79)
Aztreonam	80	53.62	0.72 (0.60-0.84)	0.77 (0.67-0.87)	0.74 (0.61-0.87)	0.70 (0.60-0.81)
Gentamicin	153	46.46	0.69 (0.55-0.80)	0.78 (0.69-0.87)	0.83 (0.73-0.91)	0.76 (0.68-0.83)
Meropenem	154	33.46	0.72 (0.58-0.84)	0.81 (0.71-0.91)	0.92 (0.86-0.96)	0.87 (0.81-0.92)
Sulfamethoxazole	100	50.59	0.75 (0.61-0.87)	0.80 (0.69-0.90)	0.80 (0.67-0.91)	0.76 (0.66-0.86)
Cefuroxime	66	63.39	0.84 (0.72-0.93)	0.90 (0.80-0.98)	0.83 (0.66-0.97)	0.75 (0.62-0.88)
Cefoxitin	66	33.93	0.60 (0.39-0.78)	0.79 (0.60-0.96)	0.92 (0.81-0.99)	0.82 (0.74-0.89)
Cefotaxime	66	61.61	0.83 (0.69-0.93)	0.87 (0.78-0.96)	0.80 (0.63-0.94)	0.75 (0.61-0.88)
Ampicillin	53	96.84	0.99 (0.99-0.99)	0.98 (0.97-1.00)	0.50 (0.13-0.88)	0.63 (0.30-0.76)
Ampicillin/Sulbactam	83	67.83	0.78 (0.67-0.87)	0.86 (0.77-0.94)	0.73 (0.52-0.89)	0.61 (0.48-0.74)
Ceftriaxone	32	63.49	0.85 (0.66-0.98)	0.92 (0.77-0.98)	0.86 (0.58-0.97)	0.77 (0.57-0.95)
Minocycline	39	22.64	0.75 (0.25-0.94)	0.84 (0.64-0.96)	0.96 (0.88-0.99)	0.93 (0.82-0.98)
Tigecycline	23	12.50	0.58 (0.17-0.83)	0.76 (0.49-0.83)	0.97 (0.97-0.98)	0.94 (0.89-0.98)
Fosfomycin	31	23.08	0.19 (0.05-0.50)	0.36 (0.08-0.82)	0.90 (0.77-0.98)	0.79 (0.74-0.86)
Median	93 (66-153) ^f^	42.90 (33.58-57.28)	0.73 (0.59-0.84)	0.79 (0.68-0.91)	0.86 (0.76-0.94)	0.78 (0.70-0.86)

a Resistance levels among all gram-negative organisms.

b Sensitivity: ability of a prior culture to detect all those with future resistance (see formulas in **Figure 1**).

c PPV: probability of a prior resistant culture to accurately predict future resistance.

d Specificity: ability of a prior culture to detect all those with future susceptibility.

e NPV: probability of a prior susceptible culture to accurately predict future susceptibility.

f Calculated the median of the median number of days between cultures for each antibiotic and the median of the first and third quartiles for each antibiotic.

### 3.3 Bayes’ NPVs and specificity

Our data revealed that relying on a prior susceptible culture had good predictability of future susceptibility (Bayes’ NPV ≥80%) for 8 antimicrobial agents spanning 4 antimicrobial categories (carbapenem, cephalosporin, aminoglycoside, and tetracyclines and derivatives). The median value of Bayes’ NPV for all antimicrobial agents was 0.78, with a median lower and upper bounds of the 95% CI ranging from 0.70 to 0.86. Bayes’ NPV was particularly high (≥85%), with a 95% CI not lower than 0.80 for the following agents: tigecycline, minocycline, amikacin, tobramycin, imipenem and meropenem. The Specificity was similarly high: the median was 0.86 (95% CI: 0.76–0.94) across all 24 tested agents and 0.93 (95% CI: 0.86–0.98) for the 8 agents.

### 3.4 Bayes’ PPVs and sensitivity

The median values of Bayes’ PPV and sensitivity across the 24 evaluated antimicrobial agents were 0.79 (95% CI: 0.68–0.91) and 0.73 (95% CI: 0.59–0.84), respectively. The probability that a prior resistant culture would predict future resistance (Bayes’ PPV) was highest (≥80%) for the following agents: ampicillin, ceftriaxone, cefuroxime, levofloxacin, ciprofloxacin, cefotaxime, ampicillin/sulbactam, tobramycin, minocycline, meropenem, and sulfamethoxazole. Meanwhile, relying on a prior resistant culture to identify future resistance (sensitivity) was ≥75% for 11 antimicrobial agents, including ampicillin, ceftriaxone, cefuroxime, cefotaxime, tobramycin, ciprofloxacin, levofloxacin, ampicillin/sulbactam, colistin, sulfamethoxazole, and minocycline.

### 3.5 Stratified sensitivity analyses of predictive performance

To assess the robustness of the predictive performance under different epidemiological contexts, stratified analyses were conducted by pathogen species (*Klebsiella pneumoniae* and *Pseudomonas aeruginosa*), time interval between paired cultures (<60 days, 61–120 days, >120 days), unit and calendar year (2019-2022). The detailed results are presented in **Supplementary Tables S1-S5**.

Across all stratified settings, the overall predictive framework remained broadly conserved and aligned with the primary analysis. Although absolute PPV and NPV values showed some fluctuation between subgroups, the relative performance pattern of antibiotic classes was largely preserved. In particular, selected β-lactams (including cephalosporins and β-lactams/β-lactamase inhibitor combinations) and fluoroquinolones repeatedly demonstrated relatively higher PPVs, whereas aminoglycosides, tetracyclines and carbapenems more often exhibited higher NPVs across the majority of stratified scenarios. Notably, this trend was observable across species-specific, interval-specific, and year-specific analyses, with similar clustering of higher-performing agents maintained throughout, reflecting stable predictive behavior under differing stratification conditions.

## 4 Discussion

In the study cohort, patients aged ≥65 years constituted the majority (70.00%), and the incidence of RRTI increased with advancing age, a trend aligning with prior studies (Rafeq and Igneri, 2022; Torres et al., 2021). Limited data are available on bacterial pathogens and antibiotic resistance specific to RRTIs. While some studies identified *Streptococcus pneumoniae* as the predominant pathogen in RTIs, our findings revealed a lower prevalence of this organism and a higher incidence of Gram-negative bacteria, particularly *Pseudomonas aeruginosa* and *Klebsiella pneumoniae*. This difference may be attributed to our predominantly elderly, hospitalized cohort as well as to geographic variations. The shift in pathogen distribution observed in our study is supported by recent regional data. Recent evidence from China indicates that the isolation rates of *Pseudomonas aeruginosa* and *Klebsiella pneumoniae* in RTIs increase with advancing age (Li et al., 2021). Additionally, Asia-Pacific reports document a rising prevalence of Gram-negative bacteria in RTIs, particularly dominated by *Pseudomonas aeruginosa* (Yang et al., 2025).

These Gram-negative pathogens are commonly associated with multidrug resistance (Satlin et al., 2023). Our study revealed concerning antimicrobial resistance patterns in respiratory pathogens isolated from patients with RRTIs. *Pseudomonas aeruginosa* exhibited marked resistance to aztreonam (52.3%) and colistin (63.1%). The latter is particularly concerning as colistin serves as a last-resort agent for multidrug-resistant infections when conventional therapies fail. Furthermore, most tested β-lactams—including piperacillin (36.9%), piperacillin-tazobactam (32.7%), cefoperazone/Sulbactam (30.6%), and carbapenems (meropenem 33.3%; imipenem 32.4%)—and fluoroquinolones (ciprofloxacin 39.6%; levofloxacin 44.7%), fosfomycin (30.3%) demonstrated resistance rates exceeding 30%. Similarly, *Klebsiella pneumoniae* showed universal ampicillin resistance (100%), with high-level resistance (>40%) to 13 tested agents. The convergence of fluoroquinolones resistance (ciprofloxacin 51.4%; levofloxacin 50.0%) and colistin resistance (46.9%) in *Klebsiella pneumoniae* with the corresponding resistances in *Pseudomonas aeruginosa* in these elderly patients indicated shared evolutionary pressures driving multidrug resistance in Gram-negative pathogens. These higher resistance rates likely result from frequent antibiotic exposure and the high comorbidity burden among older adults. According to the CHINET Bacterial Resistance Surveillance Technical Protocol and the WHO Global Antimicrobial Resistance and Use Surveillance System Report, antibiotics with resistance rates exceeding 50% should not be used empirically. For antibiotics with resistance rates between 20% and 50%, empirical use requires a rigorous risk assessment of infection severity, with priority given to alternative antibiotics. Although current guidelines recommend β-lactams or fluoroquinolones for initial empirical treatment of community-acquired pneumonia (Moja et al., 2024; Raghav et al., 2022), our data indicate high resistance to these agents in RRTIs, rendering them unsuitable for empirical therapy in this specific patient population.

The significant resistance of these pathogens to antibiotics complicates the treatment of RRTIs, making it crucial to recognize risk factors and initiate appropriate empirical therapy. However, the existing literature contains few studies on the prediction of antibiotic resistance in RRTIs. This study used a Bayesian prevalence-calibrated estimation to predict antibiotic resistance and susceptibility in subsequent hospitalizations based on prior culture results, guiding empirical antibiotic selection when current culture results are unavailable. Our findings demonstrated that prior resistance predicted future resistance with >70% accuracy (Bayesian PPV) for 21 antimicrobials, including cephalosporins, penicillins, carbapenems, β-lactamase inhibitor combinations, sulfamethoxazole, colistin, minocycline, and aztreonam. Notably, prior resistance to ciprofloxacin, levofloxacin, cefuroxime, cefotaxime, ampicillin, ampicillin-sulbactam, and ceftriaxone achieved highly predictive accuracy (≥85% PPV). These drugs with high resistance prediction accuracy (e.g., fluoroquinolones, cephalosporins, ampicillin) are often clinically important and raise significant resistance concerns. The approach’s ability to flag “persistent resistance” helps avoid therapeutic missteps. In contrast, we did not find previous susceptibility to be as good a predictor of future susceptibility. Only prior susceptibility to carbapenems, aminoglycosides, minocycline, and tigecycline predicted future susceptibility with a probability of > 0.85. This indicates that susceptibility profiles in this population are dynamic, possibly reflecting the complexity of treating RRTIs. Nevertheless, prior culture results exhibited high specificity, with 19 antimicrobials showing specificity > 0.80, reflecting a low false-positive rate and good predictive capability across diverse antibiotic classes. Additionally, over half of all antibiotics demonstrated sensitivity ≥0.7, supporting clinical safety.

Based on our findings, clinicians should avoid using antimicrobials for empirical coverage when prior cultures demonstrate resistance and the institution-specific Bayesian PPV is ≥0.80. This is particularly critical for fluoroquinolones and key β-lactams (e.g., cefuroxime, cefotaxime, ceftriaxone), where PPVs often exceed 0.85. For antimicrobials with a PPV below 0.80, empirical use may be considered with caution, guided by local susceptibility patterns and clinical context. Crucially, these PPV thresholds are institution-specific and must be derived from local resistance data; applying unvalidated, external values risk inappropriate therapy. Collectively, these findings suggest that prior culture results can help predict future resistance, informing individualized empirical therapy.

Furthermore, stratified analyses by species, interval, unit, and year showed that, although minor variations in individual antibiotics were observed, the overall predictive framework remained stable across strata: high-PPV agents continued to demonstrate reliable prediction of future resistance, while high-NPV agents consistently indicated future susceptibility. This stability across heterogeneous clinical contexts reinforces the robustness and generalizability of our approach, suggesting that prior susceptibility results can serve as a dependable reference for guiding empirical therapy decisions in diverse real-world settings. This study has several limitations. First, as the data are from a single medical center, the specific patient population and local antibiotic practices may introduce selection bias. Second, the calculated PPV and NPV are influenced by the prevalence of resistance, which may limit their generalizability to populations with differing baseline rates. Third, the lack of data on antimicrobial exposures between episodes may have affected subsequent phenotypes and predictive performance. Our prevalence-calibrated Bayesian approach intentionally yields “bespoke,” locally valid post-test probabilities appropriate for center-level decision-making, while multicenter validation remains essential to definitively assess the predictive value of prior cultures in patients with RRTIs.

## 5 Conclusion

Our retrospective study showed that RRTI patients exhibit considerable resistance to commonly used antibiotics such as ampicillin, cephalosporins, and fluoroquinolones. These findings underscore the need for bacterial cultures in RRTI patients. Applying a Bayesian prevalence-calibrated estimation to prior culture data enables prediction of future resistance, thereby providing useful information for individualized empirical antibiotic selection in this patient population.

## Data Availability

The original contributions presented in the study are included in the article/**Supplementary Material**. Further inquiries can be directed to the corresponding authors.
