# Supplementary material for: Prior culture-guided prediction of antibiotic susceptibility in recurrent respiratory tract infections: a retrospective cohort analysis
Source: Front Cell Infect Microbiol. 2026 Jan 6;15:1715986. doi: 10.3389/fcimb.2025.1715986 (PMC12816384; doi:10.3389/fcimb.2025.1715986)
Supplement: Supplementary file 1 [file DataSheet1.pdf]

Bayesian Prevalence-Calibrated Estimation: Full Technical Details

This section provides a complete description of the Bayesian prevalence-calibrated estimation used to evaluate the predictive value of a prior respiratory culture for the susceptibility phenotype of a subsequent culture. The approach does not rely on distributional priors, hyperpriors, or probabilistic sampling; instead, it combines empirically derived likelihood terms with antibiotic-specific prevalence to generate reproducible posterior PPV/NPV estimates. All computational steps, smoothing rules, and diagnostic procedures are reported to ensure full transparency and reproducibility.

1. Data structure and likelihood formulation

For each patient with ≥2 respiratory cultures, paired phenotypes were generated for each antibiotic–organism combination.

The prior culture was regarded as the test result, and the subsequent culture served as the reference standard.

	Subsequent Resistant	Subsequent Susceptible
Prior Resistant	TP	FP
Prior Susceptible	FN	TN

From these, likelihood terms are defined as:

$$Sens = \frac{TP}{TP+FN} \quad , \quad Spec = \frac{TN}{TN+FP}$$

These likelihood quantities describe how well a prior phenotype predicts a future phenotype within the same patient.

2. Priors and hyperpriors for all parameters

In this study, the Bayesian prevalence-calibrated uses a single empirical prior probability:

$$P(R) = \frac{\text{Number of resistant gram-negative isolates}}{\text{Total gram-negative isolates in the cohort}}$$

This prior reflects the antibiotic-specific resistance prevalence in the full gram-negative population. The prior is a point estimate, not a parametric Beta prior. Consequently, no hyperpriors were used. This structure matches previously published prevalence-calibrated Bayesian PPV/NPV frameworks used in clinical microbiology. Thus, the Bayesian implementation is based on empirical prevalence, not hierarchical modeling.

3. Posterior computation

Posterior predictive values were computed directly via Bayes' theorem:

Posterior positive predictive value (PPV)

$$PPV = \frac{Sens \cdot P(R)}{Sens \cdot P(R) + (1\text{-}Spec)(1\text{-}P(R))}$$

Posterior negative predictive value (NPV)

$$NPV = \frac{Spec\,(1\text{-}P(R))}{Spec\,(1\text{-}P(R)) + (1\text{-}Sens) \cdot P(R)}$$

No numerical optimization, MCMC, or iterative Bayesian sampling was required because the model is algebraically closed-form.

4. Estimation scheme and tuning choices

The computational workflow consisted of:

- Construct antibiotic-specific paired tables
- Compute likelihood terms (Sen/Spec)
- Apply Laplace smoothing to stabilize estimates
- Insert likelihood + prior into Bayes' theorem
- Use bootstrap resampling for 95% confidence intervals

5. Laplace smoothing rule (α) and rationale

Laplace correction was applied to avoid extreme estimates in small-sample or zero-cell situations:

$$Sens' = \frac{TP+\alpha}{TP+FN+2\alpha}, \quad Spec' = \frac{TN+\alpha}{TN+FP+2\alpha}$$

The smoothing parameter α followed a predefined rule based on sample size:

Condition	α value
Condition α value ≥30 observations in both Sen & Spec denominators	0.1
10–29 observations	0.5
Any zero cell or <10 observations	1.0

6. Convergence and diagnostic criteria

Given the closed-form structure of the model and the use of empirical prevalence as a fixed prior, more complex hierarchical Bayesian formulations and MCMC-based posterior sampling were not required for the objectives of this study.

7. Prevalence stratification and weighting

The prior probability of resistance was:

- Antibiotic-specific
- Calculated from all gram-negative isolates, not limited to paired isolates
- Unweighted, giving each isolate equal influence

Using full-cohort prevalence ensures that posterior PPV/NPV reflect the antimicrobial resistance ecology that clinicians encounter in practice.

No time-window, species-specific, or ward-specific weighting was applied, because such stratification would reduce stability and external applicability for empiric therapy decisions.

Worked example

This example illustrates the complete workflow for one antibiotic and one organism. Values below are representative; actual values were generated directly from the study dataset.

Example antibiotic: Meropenem

Organism: G-

1. Raw paired counts

TP = 41, FN = 15, FP = 8, TN = 93

2. Likelihood terms

$$Sens = \frac{41}{41+15} = 0.73 \quad Spec = \frac{93}{93+8} = 0.92$$

3. Laplace smoothing

$$Sens' = \frac{41+0.1}{41+15+0.2} = 0.74 \quad Spec' = \frac{93+0.1}{93+8+0.2} = 0.92$$

4. Prior prevalence

$$P(R)=0.34$$

5. Posterior estimation

$$PPV = \frac{0.74 \cdot 0.34}{0.74 \cdot 0.34 + (1\text{-}0.92)(1\text{-}0.34)} = 0.83$$

$$NPV = \frac{0.92\ (1\text{-}0.34)}{0.92\ (1\text{-}0.34) + (1\text{-}0.74) \cdot 0.34} = 0.87$$

6. Bootstrap CI

- Sensitivity: 0.61–0.84
- Specificity: 0.86–0.97
- PPV: 0.73–0.92
- NPV: 0.82–0.92

Note: The counts and derived metrics in this worked example are illustrative only and do not correspond to the actual meropenem dataset analysed in this study; they are provided solely to demonstrate the computational pipeline.

Supplementary Information

Fungal

Of the fungal isolates obtained from 183 visits, *Candida albicans* (N=319, 73.67%) and *Candida glabrata* (N=77, 17.78%) accounted for the majority. All Candida albicans isolates demonstrated 100% in vitro susceptibility to the five antifungal agents tested (flucytosine, amphotericin B, fluconazole, itraconazole, and voriconazole). However, it should be emphasized that this finding merely reflects laboratory susceptibility patterns and does not justify the empirical use of antifungal agents in respiratory tract infections. In respiratory specimens, the detection of Candida species almost invariably represents colonization rather than true infection. Therefore, antifungal “susceptibility” results should not be used to guide empirical antifungal therapy in the absence of clear evidence of invasive candidiasis, such as candidemia, histopathological confirmation of tissue invasion, or radiological features consistent with invasive fungal disease, in accordance with IDSA and ESCMID recommendations.

Table S1 Test Metric Results From Comparing Paired, Pseudomonas aeruginosa by Antibiotic

Pseudomonas aeruginosa	Sensitivity (95% CI)[b]	Bayes' PPV (95% CI)[c]	Specificity (95% CI)[d]	Bayes' NPV (95% CI)[e]
Ceftazidime	0.31 (0.12-0.50)	0.38 (0.19-0.64)	0.86 (0.74-0.95)	0.81 (0.77-0.86)
Cefepime	0.40 (0.13-0.66)	0.43 (0.18-0.63)	0.81 (0.69-0.91)	0.79 (0.72-0.87)
Imipenem	0.66 (0.46-0.83)	0.60 (0.45-0.77)	0.79 (0.66-0.90)	0.83 (0.75-0.91)
Cefoperazone/Sulbactam	0.50 (0.28-0.68)	0.69 (0.49-0.89)	0.90 (0.81-0.97)	0.80 (0.74-0.86)
Piperacillin/Tazobactam	0.48 (0.24-0.70)	0.51 (0.30-0.70)	0.77 (0.64-0.89)	0.75 (0.66-0.85)
Amikacin	0.50 (0.17-0.83)	0.39 (0.22-0.68)	0.95 (0.89-0.99)	0.97 (0.95-0.99)
Tobramycin	0.50 (0.13-0.88)	0.44 (0.17-0.77)	0.93 (0.84-0.99)	0.94 (0.90-0.99)
Ciprofloxacin	0.67 (0.50-0.83)	0.75 (0.60-0.89)	0.85 (0.74-0.94)	0.80 (0.71-0.88)
Levofloxacin	0.69 (0.45-0.86)	0.78 (0.62-0.93)	0.85 (0.68-0.96)	0.77 (0.65-0.88)
Colistin	0.76 (0.61-0.88)	0.77 (0.66-0.86)	0.60 (0.38-0.78)	0.60 (0.43-0.76)
Piperacillin	0.45 (0.24-0.65)	0.49 (0.31-0.66)	0.73 (0.57-0.85)	0.69 (0.60-0.79)
Aztreonam	0.70 (0.57-0.82)	0.74 (0.63-0.85)	0.73 (0.59-0.86)	0.69 (0.59-0.80)
Gentamicin	0.48 (0.18-0.72)	0.57 (0.33-0.78)	0.88 (0.78-0.95)	0.83 (0.75-0.90)
Meropenem	0.70 (0.48-0.88)	0.72 (0.54-0.88)	0.86 (0.75-0.95)	0.85 (0.76-0.93)
Fosfomycin	0.08 (0.06-0.17)	0.16 (0.06-0.59)	0.81 (0.55-0.95)	0.67 (0.58-0.72)

Table S2 Test Metric Results From Comparing Paired, Klebsiella pneumoniae by Antibiotic

Klebsiella pneumoniae	Sensitivity (95% CI)[b]	Bayes' PPV (95% CI)[c]	Specificity (95% CI)[d]	Bayes' NPV (95% CI)[e]
Ceftazidime	0.69 (0.47-0.89)	0.88 (0.72-0.97)	0.93 (0.81-0.98)	0.80 (0.70-0.92)
Cefepime	0.64 (0.41-0.85)	0.87 (0.68-0.97)	0.93 (0.81-0.98)	0.79 (0.69-0.90)
Imipenem	0.50 (0.21-0.79)	0.66 (0.38-0.92)	0.91 (0.81-0.98)	0.85 (0.77-0.93)
Cefoperazone/Sulbactam	0.59 (0.34-0.82)	0.94 (0.88-0.96)	0.98 (0.97-0.99)	0.83 (0.74-0.92)
Piperacillin/Tazobactam	0.64 (0.41-0.85)	0.87 (0.68-0.97)	0.93 (0.81-0.98)	0.79 (0.69-0.90)
Amikacin	0.58 (0.17-0.90)	0.67 (0.33-0.90)	0.96 (0.88-0.99)	0.94 (0.89-0.99)
Tobramycin	0.94 (0.88-0.96)	0.83 (0.65-0.94)	0.85 (0.61-0.96)	0.95 (0.89-0.97)
Ciprofloxacin	0.65 (0.35-0.93)	0.93 (0.86-0.95)	0.94 (0.90-0.96)	0.72 (0.58-0.93)
Levofloxacin	0.63 (0.43-0.81)	0.88 (0.72-0.97)	0.91 (0.77-0.98)	0.71 (0.61-0.83)
Colistin	0.68 (0.45-0.89)	0.66 (0.51-0.85)	0.69 (0.48-0.89)	0.71 (0.57-0.87)
Aztreonam	0.63 (0.17-0.90)	0.81 (0.58-0.90)	0.83 (0.75-0.90)	0.66 (0.48-0.87)
Gentamicin	0.57 (0.34-0.78)	0.69 (0.53-0.87)	0.75 (0.58-0.91)	0.64 (0.52-0.78)
Meropenem	0.50 (0.21-0.79)	0.66 (0.38-0.92)	0.91 (0.81-0.98)	0.84 (0.77-0.93)
Sulfamethoxazole	0.65 (0.32-0.89)	0.75 (0.49-0.95)	0.91 (0.79-0.98)	0.86 (0.76-0.95)
Cefuroxime	0.64 (0.43-0.83)	0.87 (0.70-0.97)	0.92 (0.79-0.98)	0.76 (0.65-0.86)
Cefoxitin	0.53 (0.30-0.73)	0.75 (0.52-0.95)	0.88 (0.71-0.98)	0.73 (0.63-0.82)
Cefotaxime	0.61 (0.37-0.82)	0.79 (0.59-0.96)	0.88 (0.74-0.98)	0.75 (0.64-0.87)
Ampicillin	0.98 (0.98-0.98)	1.00 (1.00-1.00)	0.75 (0.75-0.90)	0.04 (0.03-0.05)
Ampicillin/Sulbactam	0.63 (0.43-0.82)	0.77 (0.60-0.95)	0.79 (0.58-0.96)	0.67 (0.55-0.81)
Ceftriaxone	0.61 (0.30-0.93)	0.89 (0.72-0.94)	0.95 (0.88-0.97)	0.79 (0.68-0.95)
Minocycline	0.50 (0.17-0.83)	0.73 (0.47-0.86)	0.75 (0.75-0.83)	0.53 (0.40-0.77)
Tigecycline	0.58 (0.17-0.83)	0.73 (0.45-0.81)	0.96 (0.93-0.96)	0.92 (0.85-0.97)
Fosfomycin	0.75 (0.75-0.88)	0.73 (0.67-0.79)	0.96 (0.93-0.97)	0.96 (0.96-0.98)

Table S3 Test metric results of paired cultures stratified by time interval and antibiotic

	＜60 days		61-120 days		＞120 days	
	PPV	NPV	PPV	NPV	PPV	NPV
Ceftazidime	0.79 (0.72-0.87)	0.74 (0.68-0.80)	0.75 (0.66-0.84)	0.79 (0.74-0.84)	0.72 (0.63-0.82)	0.81 (0.77-0.86)
Cefepime	0.79 (0.70-0.88)	0.72 (0.64-0.80)	0.78 (0.69-0.87)	0.74 (0.66-0.81)	0.72 (0.61-0.83)	0.79 (0.73-0.86)
Imipenem	0.75 (0.65-0.86)	0.84 (0.78-0.90)	0.78 (0.69-0.88)	0.82 (0.75-0.89)	0.69 (0.57-0.81)	0.88 (0.83-0.93)
Cefoperazone/Sulbactam	0.75 (0.65-0.86)	0.75 (0.70-0.81)	0.69 (0.58-0.81)	0.81 (0.76-0.86)	0.68 (0.57-0.81)	0.81 (0.77-0.86)
Piperacillin/Tazobactam	0.77 (0.67-0.86)	0.76 (0.69-0.83)	0.83 (0.76-0.91)	0.68 (0.59-0.77)	0.71 (0.61-0.82)	0.81 (0.75-0.87)
Amikacin	0.80 (0.67-0.94)	0.88 (0.83-0.92)	0.50 (0.34-0.79)	0.97 (0.95-0.98)	0.71 (0.56-0.90)	0.92 (0.89-0.95)
Tobramycin	0.82 (0.70-0.94)	0.91 (0.85-0.97)	0.71 (0.54-0.89)	0.95 (0.92-0.98)	0.85 (0.74-0.95)	0.90 (0.83-0.96)
Ciprofloxacin	0.87 (0.79-0.95)	0.80 (0.72-0.88)	0.86 (0.77-0.94)	0.82 (0.74-0.89)	0.90 (0.84-0.96)	0.75 (0.65-0.85)
Levofloxacin	0.90 (0.84-0.95)	0.74 (0.67-0.82)	0.88 (0.81-0.95)	0.77 (0.70-0.85)	0.90 (0.84-0.96)	0.73 (0.66-0.82)
Colistin	0.80 (0.73-0.87)	0.64 (0.54-0.75)	0.67 (0.58-0.77)	0.78 (0.70-0.85)	0.75 (0.67-0.83)	0.71 (0.61-0.80)
Piperacillin	0.44 (0.27-0.62)	0.73 (0.65-0.82)	0.48 (0.30-0.65)	0.70 (0.61-0.79)	0.51 (0.32-0.68)	0.68 (0.58-0.77)
Aztreonam	0.80 (0.72-0.89)	0.65 (0.55-0.77)	0.80 (0.72-0.89)	0.66 (0.56-0.77)	0.70 (0.60-0.83)	0.76 (0.68-0.85)
Gentamicin	0.81 (0.72-0.89)	0.72 (0.65-0.80)	0.78 (0.69-0.88)	0.75 (0.67-0.82)	0.73 (0.63-0.84)	0.80 (0.74-0.86)
Meropenem	0.81 (0.71-0.91)	0.87 (0.81-0.92)	0.86 (0.78-0.93)	0.82 (0.75-0.89)	0.78 (0.67-0.89)	0.89 (0.84-0.93)
Sulfamethoxazole	0.81 (0.70-0.91)	0.75 (0.65-0.85)	0.77 (0.66-0.89)	0.79 (0.70-0.88)	0.84 (0.75-0.92)	0.71 (0.60-0.82)
Cefuroxime	0.95 (0.90-0.99)	0.57 (0.42-0.76)	0.87 (0.76-0.98)	0.79 (0.67-0.90)	0.87 (0.76-0.98)	0.79 (0.67-0.90)
Cefoxitin	0.85 (0.69-0.97)	0.75 (0.66-0.84)	0.84 (0.68-0.97)	0.76 (0.67-0.85)	0.70 (0.48-0.94)	0.88 (0.82-0.93)
Cefotaxime	0.93 (0.88-0.98)	0.59 (0.43-0.78)	0.83 (0.72-0.94)	0.81 (0.68-0.91)	0.85 (0.74-0.95)	0.79 (0.66-0.90)
Ampicillin	1.00 (1.00-1.00)	0.05 (0.01-0.09)	0.95 (0.92-0.99)	0.85 (0.58-0.91)	0.99 (0.98-1.00)	0.52 (0.21-0.66)
Ampicillin/Sulbactam	0.92 (0.86-0.97)	0.46 (0.33-0.61)	0.82 (0.71-0.92)	0.68 (0.56-0.80)	0.83 (0.73-0.93)	0.65 (0.53-0.78)
Ceftriaxone	0.94 (0.84-0.99)	0.68 (0.47-0.93)	0.94 (0.83-0.99)	0.70 (0.49-0.94)	0.88 (0.71-0.97)	0.82 (0.66-0.97)
Minocycline	0.87 (0.70-0.96)	0.91 (0.78-0.98)	0.75 (0.50-0.92)	0.96 (0.89-0.99)	0.81 (0.60-0.95)	0.94 (0.84-0.99)
Tigecycline	0.78 (0.51-0.84)	0.94 (0.88-0.97)	0.88 (0.69-0.92)	0.88 (0.78-0.95)	0.60 (0.31-0.69)	0.97 (0.95-0.99)
Fosfomycin	0.40 (0.10-0.85)	0.76 (0.71-0.84)	0.40 (0.10-0.85)	0.76 (0.71-0.83)	0.29 (0.06-0.77)	0.84 (0.80-0.90)

Table S4. Test metric results of paired cultures stratified by calendar year and antibiotic

	2019		2020		2021		2022	
	PPV	NPV	PPV	NPV	PPV	NPV	PPV	NPV
Ceftazidime	0.72 (0.63-0.82)	0.81 (0.76-0.86)	0.83 (0.77-0.90)	0.69 (0.62-0.76)	0.78 (0.70-0.86)	0.75 (0.70-0.81)	0.65 (0.55-0.76)	0.86 (0.82-0.89)
Cefepime	0.72 (0.61-0.83)	0.79 (0.73-0.86)	0.85 (0.78-0.92)	0.63 (0.54-0.72)	0.75 (0.65-0.85)	0.77 (0.70-0.84)	0.69 (0.58-0.81)	0.81 (0.75-0.87)
Imipenem	0.79 (0.70-0.88)	0.81 (0.74-0.88)	0.75 (0.65-0.86)	0.84 (0.78-0.90)	0.64 (0.52-0.78)	0.90 (0.86-0.94)	0.75 (0.64-0.85)	0.85 (0.79-0.91)
Cefoperazone/Sulbactam	0.73 (0.62-0.84)	0.78 (0.73-0.83)	0.69 (0.58-0.81)	0.81 (0.76-0.86)	0.72 (0.62-0.83)	0.78 (0.73-0.83)	0.69 (0.58-0.81)	0.80 (0.76-0.85)
Piperacillin/Tazobactam	0.66 (0.55-0.79)	0.85 (0.79-0.90)	0.79 (0.71-0.88)	0.74 (0.66-0.81)	0.82 (0.74-0.89)	0.70 (0.62-0.79)	0.73 (0.63-0.83)	0.80 (0.73-0.86)
Amikacin	0.68 (0.52-0.89)	0.93 (0.90-0.95)	0.82 (0.70-0.94)	0.86 (0.81-0.91)	0.69 (0.54-0.89)	0.93 (0.90-0.95)	0.70 (0.54-0.90)	0.92 (0.90-0.95)
Tobramycin	0.01 (0.01-0.04)	1.00 (1.00-1.00)	0.68 (0.52-0.88)	0.96 (0.93-0.98)	0.88 (0.78-0.96)	0.87 (0.79-0.95)	0.81 (0.68-0.94)	0.92 (0.86-0.97)
Ciprofloxacin	0.87 (0.79-0.95)	0.80 (0.72-0.88)	0.94 (0.90-0.98)	0.63 (0.52-0.76)	0.88 (0.80-0.95)	0.80 (0.71-0.88)	0.88 (0.80-0.95)	0.79 (0.71-0.88)
Levofloxacin	0.91 (0.85-0.96)	0.73 (0.65-0.81)	0.93 (0.89-0.97)	0.64 (0.56-0.75)	0.85 (0.77-0.93)	0.81 (0.75-0.88)	0.89 (0.83-0.95)	0.76 (0.68-0.84)
Colistin	0.00 (0.00-0.00)	1.00 (1.00-1.00)	0.83 (0.77-0.89)	0.59 (0.49-0.71)	1.00 (1.00-1.00)	0.00 (0.00-0.01)	0.70 (0.61-0.79)	0.76 (0.67-0.84)
Piperacillin	0.31 (0.17-0.48)	0.83 (0.76-0.89)	0.74 (0.57-0.85)	0.43 (0.33-0.55)	0.38 (0.22-0.55)	0.78 (0.70-0.85)	0.52 (0.33-0.68)	0.67 (0.58-0.77)
Aztreonam	0.79 (0.70-0.88)	0.68 (0.58-0.79)	0.80 (0.72-0.89)	0.65 (0.55-0.77)	0.70 (0.60-0.83)	0.76 (0.68-0.85)	0.75 (0.66-0.86)	0.72 (0.62-0.82)
Gentamicin	0.70 (0.60-0.82)	0.82 (0.76-0.88)	0.87 (0.81-0.93)	0.62 (0.53-0.72)	0.77 (0.68-0.87)	0.76 (0.69-0.83)	0.73 (0.63-0.84)	0.80 (0.73-0.86)
Meropenem	0.83 (0.74-0.92)	0.85 (0.79-0.91)	0.82 (0.73-0.92)	0.85 (0.79-0.91)	0.75 (0.63-0.88)	0.90 (0.86-0.94)	0.83 (0.73-0.92)	0.85 (0.79-0.91)
Sulfamethoxazole	0.73 (0.60-0.86)	0.82 (0.74-0.90)	0.81 (0.70-0.91)	0.75 (0.65-0.85)	0.84 (0.75-0.92)	0.71 (0.60-0.82)	0.80 (0.70-0.91)	0.75 (0.65-0.85)
Cefuroxime	0.90 (0.81-0.98)	0.75 (0.61-0.88)	0.92 (0.84-0.99)	0.70 (0.56-0.85)	0.91 (0.82-0.98)	0.73 (0.59-0.87)	0.86 (0.73-0.97)	0.82 (0.71-0.92)
Cefoxitin	0.77 (0.57-0.96)	0.83 (0.76-0.90)	0.83 (0.66-0.97)	0.77 (0.68-0.86)	0.71 (0.49-0.94)	0.87 (0.82-0.92)	0.82 (0.64-0.97)	0.78 (0.70-0.87)
Cefotaxime	0.88 (0.79-0.96)	0.73 (0.59-0.87)	0.89 (0.81-0.97)	0.72 (0.57-0.86)	0.88 (0.79-0.96)	0.74 (0.60-0.88)	0.83 (0.72-0.94)	0.81 (0.68-0.91)
Ampicillin	1.00 (1.00-1.00)	0.05 (0.01-0.09)	0.98 (0.97-1.00)	0.65 (0.32-0.78)	0.98 (0.97-1.00)	0.63 (0.30-0.76)	0.98 (0.97-1.00)	0.68 (0.34-0.80)
Ampicillin/Sulbactam	0.82 (0.72-0.92)	0.67 (0.55-0.79)	0.92 (0.86-0.97)	0.46 (0.33-0.61)	0.84 (0.74-0.93)	0.65 (0.52-0.77)	0.84 (0.74-0.93)	0.64 (0.52-0.77)
Ceftriaxone	0.92 (0.78-0.98)	0.76 (0.57-0.95)	0.91 (0.77-0.98)	0.77 (0.58-0.95)	0.93 (0.80-0.98)	0.74 (0.54-0.95)	——	——
Minocycline	0.02 (0.01-0.07)	1.00 (1.00-1.00)	0.83 (0.62-0.95)	0.93 (0.83-0.98)	0.88 (0.71-0.97)	0.91 (0.77-0.98)	0.91 (0.78-0.98)	0.87 (0.69-0.97)
Tigecycline	0.02 (0.01-0.03)	1.00 (1.00-1.00)	0.86 (0.66-0.91)	0.89 (0.80-0.95)	0.82 (0.57-0.87)	0.92 (0.85-0.97)	0.02 (0.01-0.03)	1.00 (1.00-1.00)
Fosfomycin	0.22 (0.04-0.71)	0.88 (0.85-0.92)	0.50 (0.14-0.89)	0.68 (0.62-0.77)	0.62 (0.23-0.94)	0.53 (0.46-0.64)	——	——

— indicates that no resistance rate was available for that year.

Table S5: Test Metric Results From Comparing Paired, Gram-Negative Organisms by Antibiotic in Non-ICU Episodes

	Sensitivity (95% CI)[b]	Bayes' PPV (95% CI)[c]	Specificity (95% CI)[d]	Bayes' NPV (95% CI)[e]
Ceftazidime	0.64 (0.50-0.75)	0.71 (0.59-0.84)	0.86 (0.77-0.93)	0.82 (0.76-0.87)
Cefepime	0.70 (0.54-0.84)	0.70 (0.57-0.84)	0.84 (0.74-0.92)	0.84 (0.76-0.91)
Imipenem	0.63 (0.45-0.79)	0.59 (0.44-0.76)	0.86 (0.77-0.93)	0.88 (0.83-0.93)
Cefoperazone/Sulbactam	0.54 (0.41-0.76)	0.70 (0.59-0.83)	0.90 (0.84-0.95)	0.82 (0.77-0.86)
Piperacillin/Tazobactam	0.69 (0.54-0.82)	0.73 (0.60-0.86)	0.85 (0.76-0.93)	0.83 (0.76-0.89)
Amikacin	0.53 (0.23-0.74)	0.57 (0.37-0.90)	0.95 (0.90-1.00)	0.95 (0.92-0.97)
Tobramycin	0.75 (0.50-0.93)	0.81 (0.63-0.96)	0.95 (0.88-0.99)	0.93 (0.87-0.98)
Ciprofloxacin	0.73 (0.59-0.85)	0.87 (0.79-0.96)	0.90 (0.79-0.97)	0.78 (0.69-0.87)
Levofloxacin	0.82 (0.72-0.91)	0.88 (0.79-0.95)	0.86 (0.76-0.95)	0.80 (0.72-0.89)
Colistin	0.74 (0.61-0.85)	0.75 (0.66-0.84)	0.67 (0.52-0.81)	0.66 (0.55-0.78)
Piperacillin	0.44 (0.21-0.65)	0.54 (0.32-0.76)	0.81 (0.66-0.92)	0.74 (0.65-0.82)
aztreonam	0.69 (0.54-0.82)	0.72 (0.65-0.85)	0.74 (0.60-0.88)	0.71 (0.61-0.81)
Gentamicin	0.69 (0.51-0.83)	0.83 (0.72-0.93)	0.90 (0.82-0.96)	0.80 (0.72-0.88)
Meropenem	0.62 (0.41-0.80)	0.71 (0.52-0.88)	0.92 (0.86-0.97)	0.89 (0.84-0.94)
Sulfamethoxazole	0.79 (0.64-0.92)	0.77 (0.64-0.90)	0.79 (0.64-0.92)	0.81 (0.70-0.92)
Cefuroxime	0.88 (0.77-0.96)	0.91 (0.82-0.98)	0.85 (0.68-0.97)	0.81 (0.68-0.93)
Cefoxitin	0.66 (0.43-0.84)	0.77 (0.58-0.96)	0.89 (0.77-0.98)	0.83 (0.74-0.91)
Cefotaxime	0.88 (0.74-0.96)	0.88 (0.79-0.98)	0.82 (0.64-0.97)	0.81 (0.66-0.94)
Ampicillin	0.99 (0.99-0.99)	1.00 (1.00-1.00)	0.75 (0.75-0.90)	0.46 (0.41-0.52)
Ampicillin/Sulbactam	0.80 (0.67-0.91)	0.86 (0.75-0.95)	0.74 (0.50-0.92)	0.66 (0.50-0.82)
Ceftriaxone	0.92 (0.77-0.98)	0.97 (0.92-0.98)	0.95 (0.88-0.97)	0.87 (0.71-0.96)
Minocycline	0.63 (0.17-0.90)	0.81 (0.60-0.95)	0.92 (0.77-0.98)	0.81 (0.67-0.94)
Tigecycline	0.70 (0.63-0.88)	0.72 (0.66-0.77)	0.96 (0.94-0.97)	0.96 (0.95-0.98)
Fosfomycin	0.08 (0.05-0.17)	0.25 (0.10-0.67)	0.93 (0.81-0.98)	0.77 (0.75-0.80)
